# Trends, patterns and health consequences of multimorbidity among South Korea adults: Analysis of nationally representative survey data 2007-2016

**DOI:** 10.7189/jogh.10.020426

**Published:** 2020-12

**Authors:** Jungyeon Kim, Salmaan Keshavjee, Rifat Atun

**Affiliations:** 1 Department of Global Health and Population, Harvard T.H. Chan School of Public Health, Boston, Massachusetts, USA; 2 Department of Global Health and Social Medicine, Harvard Medical School, Boston, Massachusetts, USA

## Abstract

**Background:**

Multimorbidity is a global challenge. It is more common in the elderly and deprived populations. Health systems are not providing appropriate care for people with multimorbidity as they are focused on managing single diseases and are not oriented to effectively manage complexity of care-coordination for multimorbidity. This study aims to examine trends, disparities and consequences of multimorbidity over a 10-year period. It also aims to analyze different multimorbidity clusters and their association with quality of life.

**Methods:**

This study analyzes Korea National Health and Nutrition Examination Survey – a cross-sectional survey repeated each year of 100 000 individuals aged one or more in 192 regions of South Korea – for the 10-year period 2007-2016. This is a population-based study based on nationally representative survey data for 10 years in Korea. Our study included 68 590 adults aged 19 or more who answered questions on presence of diseases. 39 chronic conditions were included. Disease clustering by frequency, composition and number of diseases from the top 10 most common chronic conditions were used to establish patterns of multimorbidity clusters. We performed regression analyses to analyze annual trend and the prevalence of multimorbidity across socioeconomic strata. Regressions were performed to measure association between multimorbidity and unmet need, health care service utilization, sickness days, perceived health status, and EQ-5D.

**Results:**

Multimorbidity increased in the study period and was more prevalent in the elderly, females, and people with lower household income and education level. Multimorbidity was associated with increased unmet need, health care utilization and sickness days and reduced perceived health status and quality of life. Hypertension was the most common condition in individuals with multimorbidity. Reduced quality of life was associated with increasing number of chronic diseases and multimorbidity clusters which included stroke and arthritis.

**Conclusions:**

The prevalence of multimorbidity varied across socioeconomic strata, with higher levels and health consequences observed in individuals in lower socio-economic income groups. Different multimorbidity clusters had differential effect on the quality of life. Health system designs incorporating integrated care strategies for complex conditions are required to effectively manage multimorbidity and different multimorbidity clusters.

Multimorbidity is a huge burden on individuals, households and countries [1-8]. Multimorbidity leads to increased health care utilization and health care spending, and reduced quality of life [1-5,9]. Socioeconomic status is closely associated with the prevalence of multimorbidity, which is more common in the elderly and deprived populations [3-5,7].

People with multimorbidity tend to have high levels of unmet health care need and typically do not receive appropriate care [10,11]. This is partly because of single disease focus of health systems that are not designed to cope with the complexity of care-coordination for people with multimorbidity that have complex health care needs requiring management by multidisciplinary teams [12-14].

Prior studies have mostly focused on epidemiologic features of multimorbidity and its inequitable distribution across socioeconomic strata [2,3,15-17]. These studies have typically used cross-sectional data in specific geographic settings with varied study populations and diseases [2,3,15-20]. Published studies have commonly examined differences between populations with and without multimorbidity, but few have analyzed different patterns of disease compositions related to multimorbidity [15,17,18].

We present a study that uses nationally-representative yearly survey data from the Korea National Health and Nutrition Examination Survey in Korea (KNHANES) over a 10-year period to analyze trends of multimorbidity and patterns of multimorbidity based on disease clustering [21]. We also examine the relationship between multimorbidity and access to health care, health care utilization and quality of life. We analyzed the presence of different multimorbidity clusters with varied composition and frequency of diseases and the association of these clusters with access to health care, health care utilization and quality of life. As with most countries of the world, multimorbidity in South Korea is a major health challenge as its population is aging more rapidly than any other high-income country [22].

## METHODS

### Sample and data sources

We used data from KNHANES for the period 2007-2016 [21]. KHANES is a self-reported nationally-representative survey, designed and conducted by the Korean government each year. It is designed to collect information on socioeconomic status, health behaviors, health care utilization, medical conditions, physical and mental status, quality of life and nutrition conditions from approximately 10 000 individuals aged one or more, in 192 regions of South Korea [21]. KHANES is based on multistage cluster sampling, and survey participants change from year to year [21]. Survey questions are categorized for three different groups according to their stage of life: children (aged 1-11 years), adolescents (aged 12-18 years) and adults (aged 19 years or more) [21].

Our study sample included 68 590 adults aged 19 or more who answered questions asking presence of diseases. We excluded children under 18 years because most of these questions on diseases were limited to the adults. Following a review of published literature and a detailed report on multimorbidity [7,15] we included 39 chronic conditions available from the survey based on the classification from KNHANES’ guidelines [21]. We coded 39 chronic conditions into 28 after grouping myocardial infarction (MI) or angina into MI or angina, eight kinds of cancer (stomach, liver, colon, breast, cervix, lung, thyroid and other) into cancer, three kinds of vision problems (cataract, glaucoma and macular degeneration) into vision problems, and chronic hepatitis B and hepatitis C into viral hepatitis (**Table 1**).

**Table 1:** The annual weighted percent of multimorbidity and chronic conditions (2007–2016)

	2007	2008	2009	2010	2011	2012	2013	2014	2015	2016	Trend test *P*-value
Multimorbidity	19.2	20.7	20.8	12.2	11.6	11.7	14.9	14.1	22.7	23.7	0.007
Cancer	0.6	0.9	1.0	1.2	1.3	1.2	1.3	1.8	1.7	1.8	<0.001
Diabetes	5.6	5.5	5.7	5.7	6.3	5.7	7.0	6.1	6.6	7.9	<0.001
Thyroid disease	1.7	1.3	1.4	1.4	2.0	1.6	1.4	1.5	2.2	1.9	0.021
Depression	1.1	1.5	1.7	1.3	2.1	2.0	1.7	3.0	2.5	2.8	<0.001
Otitis	1.0	0.7	0.5	-	-	-	-	-	1.0	1.0	0.254
Vision problem	6.5	7.3	6.9	-	-	-	-	-	8.9	9.7	<0.001
Hypertension	13.2	14.8	15.3	16.2	15.6	16.1	15.9	15.3	18.4	19.1	<0.001
Dyslipidemia	3.2	3.8	4.6	5.3	5.4	6.2	7.4	7.9	10.1	11.4	<0.001
Stroke	1.3	1.1	1.0	0.7	0.8	0.8	1.5	1.1	1.5	1.3	0.063
MI or Angina	1.4	1.2	1.1	1.9	1.6	1.6	1.6	1.4	1.8	2.0	0.001
Hemorrhoids	1.9	2.0	2.3	-	-	-	-	-	-	-	0.307
Ulcer	1.4	0.9	1.0	-	-	-	-	-	-	-	0.128
Liver cirrhosis	0.1	0.1	0.1	0.2	0.1	0.2	0.2	0.1	0.3	0.2	0.079
TMJ	0.8	0.9	1.0	-	-	-	-	-	-	-	0.465
Hepatitis	1.6	0.8	0.5	0.5	0.4	0.8	0.8	0.4	0.8	0.6	0.013
Arthritis	8.8	9.1	8.5	9.7	7.6	7.5	8.8	7.5	10.0	9.1	0.936
Osteoporosis	3.3	3.0	4.1	-	-	-	-	-	5.3	5.3	<0.001
Backache	5.5	8.6	10.4	-	-	-	-	-	-	-	<0.001
Tuberculosis	0.2	0.1	0.1	0.1	0.1	0.1	0.1	0.1	0.1	0.0	0.075
Asthma	1.5	1.7	1.6	1.6	1.3	1.5	1.6	1.7	1.7	1.7	0.511
COPD	0.5	0.4	0.3	-	-	-	-	-	-	-	0.239
Sinusitis	1.8	2.3	1.7	-	-	-	-	-	2.7	2.7	0.003
Bronchiectasis	0.2	0.2	0.1	-	-	-	-	-	-	-	0.596
Rhinitis	7.1	8.4	7.3	-	-	-	10.8	11.1	14.0	13.3	<0.001
Eczema	1.8	1.9	1.7	1.6	1.9	1.6	1.9	1.8	2.1	2.3	0.162
Anemia	3.0	2.8	2.6	-	-	-	-	-	-	-	0.428
Kidney disease	0.3	0.3	0.2	0.2	0.2	0.3	0.4	0.2	0.4	0.3	0.533
UI	0.9	1.0	0.7	-	-	-	-	-	-	-	0.288

MI – myocardial infarction, TMJ – temporomandibular joint dysfunction, COPD – chronic obstructive pulmonary disease, UI – urinary Incontinence

*Multimorbidity: presence of two or more morbidities.

### Measures and analysis

As with prior studies, we defined multimorbidity as the concurrent existence of two or more of the 28 chronic conditions in one person [3,7,15]. We used the annual survey weights provided by KHANES to examine the yearly national population estimation [21]. Using this annual survey weight, we provided descriptive statistics to summarize the evolution of multimorbidity and chronic conditions and conducted logistic regression to test the linear trend of annual prevalence of multimorbidity.

Chronic conditions included in the survey questions may change over years. To minimize this potential selection bias of yearly change in chronic conditions we created a pooled weight of ten years based on the annual weights.

Based on this pooled weight we analyzed the distribution of multimorbidity across socioeconomic strata. To analyze differences in multimorbidity by socioeconomic status we first used descriptive statistics, including a box plot and histograms to visualize the distribution of multimorbidity across socioeconomic strata. We conducted bivariate and multivariate logistic regression between the prevalence of multimorbidity and socioeconomic status (age, sex, household income, education). We treated age variable as a continuous variable, sex variable as a binary variable, household income variable as a categorical variable based on household income quartiles, and education income variable as a categorical variable. We quantified the association between socioeconomic status and the prevalence of multimorbidity by reporting unadjusted and adjusted odds ratios (ORs).

We used unmet need, outpatient utilization, inpatient utilization, sickness days, perceived health status, and EQ-5D index scores as the measures of health consequences related to multimorbidity. Unmet need was a binary variable indicating that respondents have had unmet need over the past one year or not, outpatient utilization was a binary variable indicating that respondents have had outpatient visits over the past two weeks, inpatient utilization was a binary variable indicating that respondents have had inpatient visits over the past one year and sickness days was coded as a binary variable indicating that respondents have had sickness days over the past one year. Perceived health status was an ordinal variable with five categories ranging from 1-very poor to 5-very good. We treated perceived health status as continuous variable. EQ-5D [23-25] is a standardized instrument that measures five dimensions of mobility, self-care, usual activities, pain/discomfort, and anxiety/depression. EQ-5D index scores indicates health-related quality of life (HRQoL) on a scale from 0 (dead) to 1 (perfect health). We conducted logistic regressions and calculated odds ratios (OR) with 95% Confidence Intervals (CI) for the association between the presence of multimorbidity and unmet need, outpatient utilization, and inpatient utilization. We conducted regression and calculated regression coefficients with 95% confidence interval (CI) for the association between the presence of multimorbidity and perceived health status and EQ-5D index scores.

We sought to examine the extent and severity of multimorbidity, for which there is no agreed classification, by analyzing the composition and the number of diseases in individuals with multimorbidity. We considered individuals with different multimorbidity profiles differently. For example, people having stroke and depression and people having hypertension and sinusitis have different nature and amount of disease burden.

We coded the number of morbidities that are equal or greater than five as 5+ and we analyzed the most common ten combinations of morbidities per each number of morbidities (1, 2, 3, 4, 5+). We coded the remaining combination of morbidities as ‘Other.’ We examined the relationship between EQ-5D index number (utility)[23-25] and the top ten most common composition of morbidities and ‘Other’ composition per number of morbidities. To analyze different profiles of multimorbidity clusters, we examined the composition of multimorbidity, the frequency of these compositions across the number of morbidities and the effect of the number of morbidities and the composition of morbidity clusters on the quality of life.

## RESULTS

### Descriptive statistics

The prevalence of multimorbidity increased from 19.2% in 2007 to 23.7% in 2016 (**Table 1**). Among the morbidities included in our study, cancer, diabetes, thyroid disease, depression, vision problems, hypertension, dyslipidemia, MI or angina, hepatitis, arthritis, osteoporosis, backache, sinusitis, and rhinitis showed statistically significant different annual trends for the ten years of the study period (**Table 1**).

### The relationship between multimorbidity and age, sex, household income, and education

**Figure 1:**
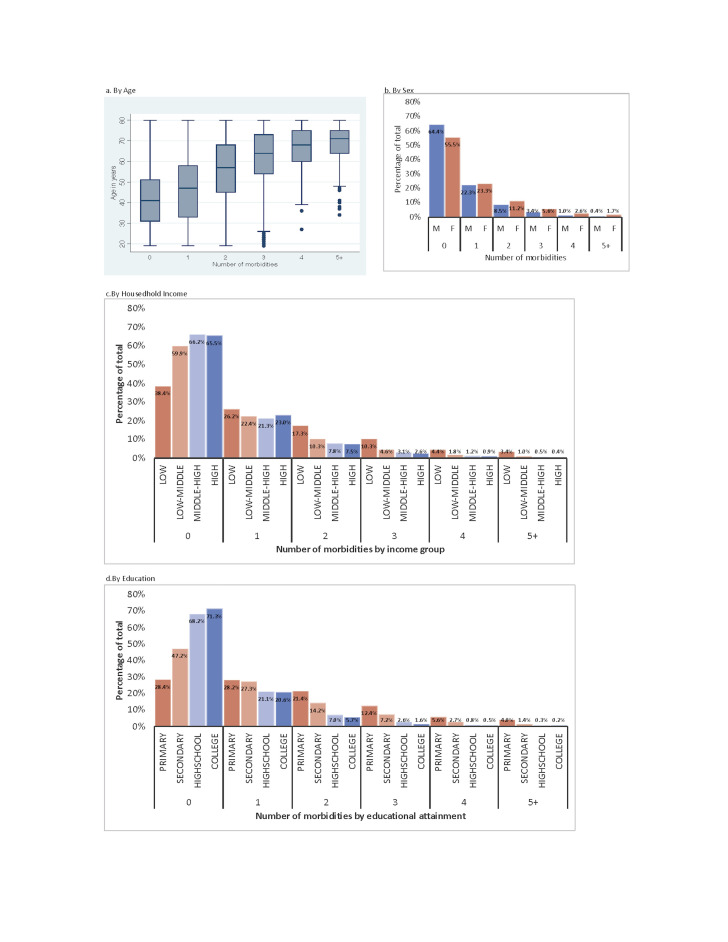
Disparities of multimorbidity across socioeconomic strata.

**Figure 1** shows the distribution of multimorbidity across socioeconomic strata by number of morbidities. The number of morbidities increased with the age (**Figure 1**, Panel A). The mean age of healthy people without any morbidity was 39.9 years (95% CI = 39.7, 40.2), while the mean age of people having four morbidities was 65.2 years (95% CI = 64.4, 66.0) (**Figure 1**, Panel A). Approximately 21.1% of females (95% CI = 20.6, 21.7) and 13.3% of males (95% CI = 12.7, 13.8) (**Figure 1**, Panel B) had multimorbidity.

The prevalence of multimorbidity nearly tripled in low-income households (first quartile) (35.4%, 95% CI = 34.1, 36.6) compared to high-income households (fourth quartile) (11.5%, 95% CI = 10.9, 12.1) (**Figure 1**, Panel C). Number of morbidities increased with decreasing education level (**Figure 1**, Panel D). 43.4% (95% CI = 42.3, 44.5) of people who attained primary education, 25.5% (95% CI = 24.2, 26.9) of people who attained secondary education, 10.7% (95% CI = 10.2, 11.2) of people who attained high school education, and 8.1% (95% CI = 7.6, 8.6) of people who attained college education had multimorbidity (**Figure 1**, Panel D).

**Table 2** shows the association between socioeconomic status and multimorbidity. For a one-year increase in age the odds of having multimorbidity increased by a factor of 1.070 (95% CI = 1.067, 1.073. For females the odds increased by a factor of 1.520 (95% CI = 1.434, 1.610) compared to males (**Table 2**). The OR of having multimorbidity was 0.917 (95% CI = 0.889, 0.945) for one-unit increase in household income and 0.872 (95% CI = 0.845, 0.900) for one-unit increase in education level (**Table 2**). The effect sizes became larger when we compared individuals without any condition to individuals with multimorbidity (**Table 2**).

**Table 2:** Association between socioeconomic status and multimorbidity

	Unadjusted			Adjusted		
	**n**	**Odds ratio**	**95% CI**	**n**	**Odds ratio**	**95%CI**
**When comparing to people with single morbidity or without any morbidity:**						
Age	56 970	1.080	1.077 1.082	55 630	1.070	1.067, 1.073
Sex (Female)	56 970	1.754	1.670 1.842	54 956	1.520	1.434, 1.610
Household Income (4)	56 240	0.617	0.600 0.634	54 956	0.917	0.889, 0.945
Education (4)	56 332	0.454	0.442 0.466	54 956	0.872	0.845, 0.900
**When comparing to healthy people without any morbidity:**						
Age	43 177	1.101	1.098 1.104	42 154	1.088	1.085, 1.092
Sex (Female)	43 177	1.850	1.758 1.948	41 633	1.581	1.480, 1.689
Household Income (4)	42 627	0.581	0.563 0.599	41 633	0.882	0.852, 0.914
Education (4)	42 680	0.389	0.377 0.400	41 633	0.844	0.815, 0.875

CI – confidence interval

### The relationship between multimorbidity and consequences

**Table 3** summarizes the relationship between multimorbidity and access to health care, utilization and quality of life. The odds of experiencing unmet need for people with multimorbidity was 1.490 times (95% CI = 1.386, 1.601) that of people without multimorbidity after adjusting for other covariates. People with multimorbidity were more likely to use outpatient services, with an OR of 2.190 (95% CI = 2.063, 2.324) and inpatient services with an OR of 1.634 (95% CI = 1.508, 1.771) compared to those without multimorbidity after adjusting for other covariates (**Table 3**).

**Table 3:** Relationship between multimorbidity and consequences

	Unadjusted				Adjusted			
**n**	**Odds ratio**	**95% CI**		**n**	**Odds ratio**	**95% CI**	
**A) Unmet need**								
Multimorbidity	56 472	1.451	1.362, 1.546		55 617	1.490	1.386, 1.601	
Age	56 472	0.998	0.998, 1.003			0.983	0.981, 0.985	
Sex (female)	56 472	1.651	1.559, 1.750			1.548	1.460, 1.641	
Household income (4)	55 764	0.864	0.840, 0.889			0.904	0.877, 0.933	
Education (4)	56 317	0.866	0.843, 0.889			0.833	0.803, 0.863	
**B) Outpatient utilization**								
Multimorbidity	56 402	3.071	2.914, 3.237		55 605	2.190	2.063, 2.324	
Age	56 402	1.025	1.024, 1.027			1.013	1.011, 1.015	
Sex (female)	56 402	1.525	1.460, 1.593			1.369	1.307, 1.433	
Household income (4)	55 698	0.843	0.824, 0.862			0.983	0.959, 1.008	
Education (4)	56 304	0.729	0.715, 0.744			0.925	0.900, 0.951	
**C) Inpatient utilization**								
Multimorbidity	56 407	1.734	1.616, 1.861		55 605	1.634	1.508, 1.771	
Age	56 407	1.006	1.004, 1.008			0.997	0.994, 0.999	
Sex	56 407	1.303	1.223, 1.387			1.242	1.164, 1.327	
Household income (4)	55 704	0.917	0.889, 0.946			0.977	0.944, 1.010	
Education (4)	56 303	0.874	0.849, 0.900			0.929	0.893, 0.968	
**D) Sickness days**								
Multimorbidity	56 444	2.265	2.093, 2.452		55,606	2.121	1.923, 2.339	
Age	56 444	1.007	1.004, 1.009			0.986	0.983, 0.990	
Sex (female)	56 444	2.090	1.923, 2.272			1.891	1.737, 2.059	
Household income (4)	55 736	0.760	0.733, 0.789			0.824	0.791, 0.858	
Education (4)	56 306	0.786	0.759, 0.813			0.865	0.824, 0.909	
	**Unadjusted**					**Adjusted**		
	**n**	**Coefficient**	**95% CI**		**n**	**Coefficient**	**95% CI**	
**E) Perceived health status** (1: very poor – 5: very good)								
Multimorbidity	56 518	-0.656	-0.679, -0.634		55 609	-0.493	-0.517, -0.469	
Age	56 523	-0.011	-0.012, -0.011			-0.002	-0.003, -0.001	
Sex (female)	56 523	-0.190	-0.207, -0.172			-0.116	-0.133, -0.010	
Household income (4)	55 813	0.143	0.134, 0.152			0.066	0.056, 0.075	
Education (4)	56 310	0.187	0.179, 0.194			0.077	0.067, 0.087	
**F) EQ-5D index**								
Multimorbidity	56 399	-0.101	-0.105, -0.097		55 583	-0.058	-0.062, -0.054	
Age	56 399	-0.002	-0.003, -0.002			-0.001	-0.001, -0.001	
Sex (female)	56 399	-0.031	-0.033, -0.029			-0.018	-0.020, -0.016	
Household income (4)	55 693	0.028	0.026, 0.029			0.012	0.011, 0.013	
Education (4)	56 283	0.038	0.037, 0.039			0.015	0.014, 0.017	

CI – confidence interval

We found a statistically significant relationship between multimorbidity and quality of life. The odds of being sick for people with multimorbidity increased by 112% (OR: 2.121, 95% CI = 1.923, 2.339) compared to that for people without multimorbidity, after adjusting for other covariates (**Table 3**). For people with multimorbidity, the predicted health status was lower by approximately 0.49 points (95% CI = -0.517, -0.469) and the EQ-5D index number was lower by approximately 0.06 points (95% CI = -0.062, -0.054) than for people without multimorbidity (**Table 3**).

### Health-related quality of life and diseases clusters of multimorbidity

**Figure 2:**
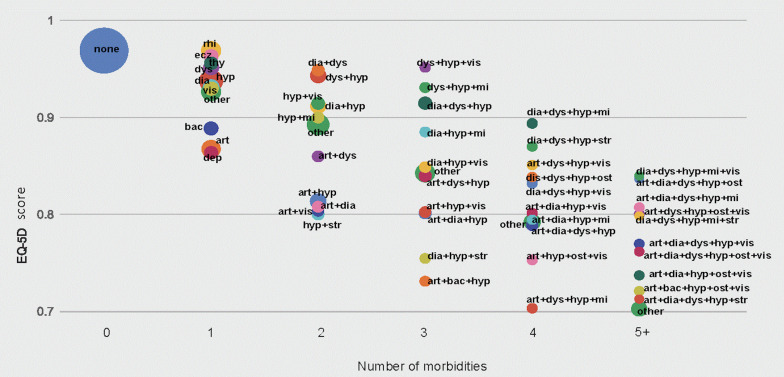
Profiles of multimorbidity and quality of life. Abbreviations: art: arthritis, bac. backache, dep: depression, dia: diabetes, dys: dyslipidemia, ecz: eczema, hyp: hypertension, mi: myocardial inforction or angina, ost: osteoporosis, rhi: rhinitis, thy: thyroid disease, vis: vision problems. Note. EQ-5D 1 = full health, 0 = death. Bubble size shows the frequency of combination as % of the total.

**Figure 2** shows the relationship between different profiles of multimorbidity by composition of conditions and the frequency and HRQoL score as measured by EQ-5D. The more conditions individuals had, the less was HRQoL (**Figure 2**). The mean of EQ-5D was 0.97 for healthy individuals, who accounted for 53% of the study sample, whereas that was 0.75 for individuals with multimorbidity living with five or more conditions, who accounted for 2% of the study sample (**Figure 2**).

The ten most common conditions among individuals having a single morbidity were hypertension (7%), rhinitis (4%), arthritis (3%), diabetes (2%), dyslipidemia (1%), vision problems (1%), backache (1%), eczema (1%), thyroid disease (1%), and depression (1%). The means of EQ-5D for these ten conditions ranged from 0.86 for individuals with depression to 0.97 for individuals with rhinitis (**Figure 2**).

There was wide range of HRQoL scores among individuals with multimorbidity depending on the composition of morbidities. Among individuals with multimorbidity living with two conditions, HRQoL was the highest for individuals living with diabetes and dyslipidemia with EQ-5D score of 0.95, and the lowest for individuals living with hypertension and stroke with EQ-5D score of 0.80.

Among individuals with multimorbidity living with three conditions, EQ-5D scores ranged from 0.73 for individuals living with arthritis, backache, and hypertension to 0.85 for those living with dyslipidemia, hypertension and vision problems (**Figure 2**).

The most common combinations of conditions for individuals with multimorbidity were arthritis and hypertension among individuals with two chronic conditions; diabetes, dyslipidemia and hypertension among those with three conditions; arthritis, diabetes, dyslipidemia and hypertension among those with four conditions, and; arthritis, dyslipidemia, hypertension, osteoporosis and vision problems among those with five or more conditions.

## DISCUSSION

In this study, we sought to advance the understanding of the level and distribution of multimorbidity among different population groups, stratified by age, sex and socioeconomic status, and provide new empirical evidence at the population level based on an analysis of pooled cross-sectional data that used nationally-representative surveys undertaken each year over a 10-year period.

We analyzed the evolution of the prevalence and distribution of multimorbidity across age, sex and socioeconomic status. We found an increasing trend of multimorbidity and substantial disparities in multimorbidity across age, sex, household income, and education level.

We also analyzed the relationship between multimorbidity and access to health care services, health utilization and quality of life. Our findings show statistically significant negative effects of multimorbidity on access to health care services and quality of life..

One of earlier study from KHANES showed that multimorbidity lowered EQ-5D scores [26]. This study extends earlier studies by providing evidence on different patterns of multimorbidity clusters and their effects on HRQoL. We found a wide spectrum of HRQoL among multimorbid individuals depending on the number of conditions and the composition of conditions.

As per earlier studies, the results of our study showed that the odds of multimorbidity increase for older people, females, individuals with low-income, and individuals with low level of education [3,4,6,18,20]. The number of co-occurring conditions increased with age and for females but decreased with an increase in income and education level.

Although people with multimorbidity were more likely to use outpatient and inpatient services, they were more likely to have unmet need for health care services. The findings suggest suboptimal management of multimorbidity despite high utilization of health care services and high levels of out-of-pocket costs incurred. People with low socioeconomic status were more likely to have multimorbidity, and experience higher risk of financial burden as a result of multimorbidity [27].

The current definition of multimorbidity is the presence of multiple coexistences of diseases within a person, and measured by counting number of diseases an individual has [7,16,28]. Based on this uni-dimensional definition, earlier studies have focused on the relationship between the presence of multimorbidity and its impact on health outcomes [1,7,10,11,29,30]. However, these studies have not explored different profiles of multimorbidity due to different combinations of diseases and how these combinations lead to different multimorbidity clusters. Clinical decisions for multimorbid patients are complex and challenging [13,31] and therefore, understanding different profiles of multimorbidity is essential for managing multimorbidity more effectively and efficiently in health systems. However, little attention has been given to understanding the features of different profiles of multimorbidity [13]. For this reason, we identified and visually presented different profiles of multimorbidity by common combinations of conditions and the number of conditions. We found multimorbidity was heterogeneous in many ways, including the number of conditions, the composition of conditions, frequency of conditions and the extent of severity as measured by HRQoL. Hypertension was one of the most common conditions for multimorbidity. The number of conditions, as well as the composition of conditions, affected HRQoL. Multimorbidity with stroke, myocardial infarction or arthritis impacted the quality of life most negatively (**Figure 2**).

When developing clinical guideline to manage patients with multimorbidity one should consider common conditions that lead to multimorbidity and the way these cluster. Like a previous study conducted in the elderly [18], hypertension was the most common single condition in patients with multimorbidity. The combination of hypertension and arthritis was the most frequent coupling among individuals with two conditions, the combination of hypertension, diabetes, and dyslipidemia was the most frequent mix among those with three conditions, and the combination of hypertension, arthritis, diabetes, and dyslipidemia was the most frequent mix among those with four conditions.

To effectively manage multimorbidity, policy makers should develop targeted policies that take into account the frequency and mix of conditions that lead to multimorbidity and different multimorbidity clusters which have varied effects on utilization of health services and the quality of life [29,32,33]. However, in practice, effective management of health systems, health care utilization and outcomes for patients with multimorbidity who are frequent users of health care services is a challenging task, as health systems are designed to manage single diseases [10,14]. Therefore, priority setting and system design should consider varying multimorbidity profiles as well as the disparities among patients with multimorbidity in different socioeconomic strata in relation to access, utilization and outcomes, for example by introducing early interventions for low-income households, including medical aid program [34], conditional cash transfers, food and nutrition assistance [35].

### Limitations

Our study has several limitations and strengths. We used self-report national survey data. Self-reported survey data are prone to potential recall bias and selection bias. However, self-reported survey data more accurately reflect the presence of multimorbidity because they are more likely to capture symptoms of chronic conditions compared to electronic health records that might be incomplete [36]. Another limitation is that the list of conditions included in the survey was not the same throughout our study period. In order to minimize potential bias, we pooled 10-year longitudinal national survey data and used 10-year pooled sample weight to estimate prevalence of multimorbidity. We sought to include all available conditions after reviewing the list of diseases included in other multimorbidity studies [15]. We also provided an annual prevalence of multimorbidity based on each year's sample weight to compare annual differences. While most studies use single-year cross-sectional data, we used nationally representative survey data that produced a 10-year longitudinal data set, enabling us to examine multimorbidity patterns at population level.

## CONCLUSIONS

Multimorbidity is increasing in high-income countries. Multimorbidity negatively affects unmet need, health care utilization, and quality of life, which affect lower socioeconomic income population groups disproportionately, with widening disparities in the prevalence, health care service utilization, HRQoL and level of financial burden over time. The composition, frequency, and the extent of multimorbidity varies widely among different age groups and socioeconomic strata. Varied combinations of conditions lead to different multimorbidity profiles. The effect of these different multimorbidity clusters on health care utilization, HRQoL and level of financial burden vary significantly. Clinical decisions of multimorbid patients is complex and challenging because health systems are designed to manage single-morbid patients. Future research is needed to develop integrated care strategies to target population groups with different profiles of morbidities to ensure effective management and prevention of multimorbidity and its consequences on health outcomes, health related quality of life and financial burden on individuals.
